# Artificial Intelligence Dystocia Algorithm (AIDA) for Risk Stratification of Occiput Posterior Fetal Head Position

**DOI:** 10.3390/jimaging12060230

**Published:** 2026-05-27

**Authors:** Antonio Malvasi, Giorgio Maria Baldini, Tommaso Difonzo, Iris Cara, Marco Cerbone, Miriam Dellino, Antonella Vimercati, Ilenia Mappa, Giuseppe Rizzo, Andrea Tinelli, Ettore Cicinelli, Edoardo Di Naro, Lorenzo E. Malgieri

**Affiliations:** 1 Unit of Obstetrics and Gynecology, Department of Interdisciplinary Medicine (DIM), University of Bari “Aldo Moro”, Policlinico of Bari, 70124 Bari, Italy; antoniomalvasi@gmail.com (A.M.); difonzo.tommaso.md@gmail.com (T.D.); carairis95@gmail.com (I.C.); marcocerbone@gmail.com (M.C.); miriam.dellino@uniba.it (M.D.); antonellavimercati@gmail.com (A.V.); ettore.cicinelli@uniba.it (E.C.); dinaroedoardo@gmail.com (E.D.N.); 2 Department of Maternal and Child Health and Urological Sciences, University of Roma Sapienza, 00185 Rome, Italy; mappa.ile@gmail.com (I.M.); giuseppe.rizzo@uniroma1.it (G.R.); 3 Department of Obstetrics and Gynaecology and CERICSAL (CEntro di Ricerca Clinico SALentino), Veris delli Ponti Hospital Scorrano, 73020 Lecce, Italy; andreatinelli@gmail.com; 4 The New European Surgical Academy (NESA), 10117 Berlin, Germany

**Keywords:** Artificial Intelligence Dystocia Algorithm (AIDA), occiput posterior position, machine learning, intrapartum ultrasound, labor dystocia, persistent occiput posterior, risk stratification

## Abstract

The occiput posterior (OP) fetal head position is the most common malposition during labor and is associated with prolonged labor, operative delivery, and cesarean section. Conventional assessment often relies on digital examination, and the clinical significance of OP may lie along a spectrum rather than as a binary diagnosis. The Artificial Intelligence Dystocia Algorithm (AIDA) integrates four objective intrapartum ultrasound parameters (Angle of Progression [AoP], Head–Symphysis Distance [HSD], Midline Angle [MLA], and Asynclitism Degree [AD]) into a five-class ordinal classification (Classes 0–4). This investigation is a focused secondary subgroup analysis of 79 OP cases drawn from a single-cohort dataset of 135 nulliparous women with prolonged second-stage labor originally collected for the development of the AIDA. Only Branch 1 of the AIDA (the deterministic threshold-based classification, with cut-offs originally derived via Decision Tree on the parent cohort, N = 135) was applied; Branch 2 (the case-level machine-learning predictors) was not used, and no predictive model was trained or validated in this study. Cesarean delivery rates rose monotonically across AIDA classes, from no cesareans in Class 0 to all cases delivering by cesarean in Class 4, with a clear gradient across intermediate classes; full numerical results, confidence intervals, and effect sizes are reported in the Results section. Because the AIDA thresholds were derived from the same parent cohort, the analysis is best interpreted as a within-cohort subgroup evaluation rather than as independent validation. The observed class-graded outcome distribution is consistent with the hypothesis that in OP labors, the AIDA class assignment itself may carry clinically relevant information on the risk of intrapartum cesarean delivery; this remains hypothesis generating, and confirmation in independent prospective cohorts is required before AIDA-class assignment can be regarded as an established risk-stratification descriptor in OP labors.

## 1. Introduction

Labor encompasses distinct phases, with the first stage characterized by cervical dilation and effacement and the second stage extending from complete dilation to fetal delivery [1,2]. Successful labor progression depends on multiple interrelated factors: maternal pelvic anatomy, uterine contractility, and both fetal presentation and position [3]. The fetus assumes an active role, engaging in cardinal movements to pass through the maternal pelvis [4]. When this adaptive process fails, second stage arrest may occur [1].

While the occiput anterior (OA) position is optimal, fetal malpositions, particularly the occiput posterior (OP) position, occur with notable frequency and are a primary cause of labor dystocia [5]. The OP position is the most common fetal malposition, with a prevalence reported to be between 1.8% and 8.4% at the time of delivery, though it can be much higher during the first stage of labor (33–58%) [6,7]. These positions are correlated with prolonged labor, increased rates of operative vaginal delivery, cesarean sections, postpartum hemorrhage, and severe perineal trauma [7,8,9,10]. The neonatal risks are also elevated, including lower Apgar scores and an increased need for neonatal intensive care unit (NICU) admission [9,10,11].

However, the clinical significance of an OP diagnosis may be more complex than a simple binary classification. The concept of an “occiput posterior spectrum” (ranging from a transient rotational position to a persistent, direct OP malposition) is more clinically relevant. Persistent OP (POP) position, where the fetus fails to rotate to an anterior position, presents the greatest challenge and carries the highest risk of adverse outcomes [12,13].

Historically, fetal position assessment has relied on digital vaginal examination (VE), evaluating fetal sutures and fontanels [14]. However, in cases of prolonged and dystocic labor, the presence of molding and caput succedaneum significantly compromises the accuracy of VE, particularly for diagnosing OP positions [14,15,16,17,18,19,20]. Studies have consistently shown that intrapartum ultrasound (IU) is a more reliable and reproducible method for determining fetal head position [14,16,18,21].

In the first stage, trans-abdominal (TA) imaging documents initial occipital orientation and evolution [22,23], while in the second stage, trans-perineal metrics quantify descent (AoP) and engagement (HSD) and reveal asynclitism (AD); concurrent TA views could confirm true OP/pOP [24,25]. Across randomized and observational studies, intrapartum ultrasound (IU) is more reliable than clinical examination for both position and station and improves procedural planning for instrumental birth, although composite maternal–neonatal outcomes do not inevitably change unless measurements are embedded in a structured decision framework [5,26]. These data justify routine IU before any intervention and advocate serial reassessment when progress deviates from expectations [27].

The ISUOG Practice Guidelines currently endorse IU when labor deviates from normal progression or when an obstetric intervention is planned [28].

**Figure 1:**
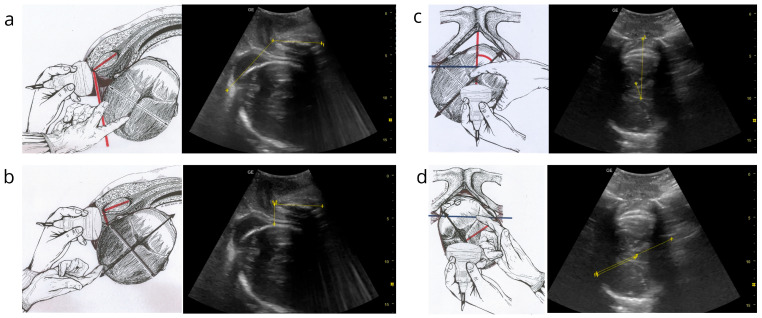
The figures illustrate the four parameters of the AIDA during the prolonged second stage of labor in occiput posterior position. (**a**) Angle of Progression (AoP): the drawing on the left (red angle) and the US photo on the right show the AoP with the fetal head in occiput posterior position (O.P.P.) (yellow angle); (**b**) Fetal Head–Symphysis Distance (HSD): the drawing on the left (red line) and the US photo on the right show the HSD (yellow line) with the fetal head in occiput posterior position (O.P.P.); (**c**) Midline Angle (MLA): the drawing on the left (red angle) and the US photo on the right (yellow angle) shows the MLA with the fetal head in the right occiput posterior position (R.O.P.P.); (**d**) Asynclitism Degree (AD): the drawing on the left (red line) and the US photo on the right (yellow short line) show the AD with the fetal head in the left occiput posterior position (L.O.P.P.). The blue transverse line indicates the biischiatic line.

Intrapartum ultrasound (IU) provides clear visualization of fetal cranial landmarks and allows for objective measurement of parameters such as the Midline Angle (MLA), which quantifies the degree of rotation [29]. This supports the diagnosis of occiput posterior (OP) and persistent OP (pOP). IU also achieves higher diagnostic accuracy for fetal head position than digital vaginal examination, particularly when caput succedaneum or molding obscure cranial sutures and fontanelles [3,4,30]. Transabdominal views are particularly valuable to identify a posterior occiput via the eyes-to-pubis sign (fetal orbits facing the pubis) and to document a completely posterior fetal spine, which together strengthen confidence in true posterior orientation prior to any intervention [23,24,31,32]. In the obstetric literature, persistent OP (pOP) has been conceptualized as a diagnosis requiring serial scans across the second stage to document sustained posterior orientation together with an unfavorable geometric signature, limited descent, and poor engagement. Defining pOP by this combination of features, rather than by the positional label alone, would identify the highest-risk end of the OP spectrum. The present study, however, is based on a single ultrasound assessment at the three-hour mark and does not include serial measurements; the framing of pOP discussed in Section 4.3 is therefore a conceptual proposal drawn from the literature, not an empirical finding of the current analysis. This interpretation is consistent with cohort data linking misdiagnosis and late recognition of OP to higher rates of failed operative vaginal delivery and intrapartum cesarean [17,33,34]. ISUOG recommends defining fetal head position using a clock face system. Positions from 3:30 to 8:30 h indicate occiput posterior position (OPP), while those from 9:30 to 2:30 h indicate occiput anterior position (OAP) [29]. Although this clock face classification is common in practice, some researchers favor an angle-based approach, especially emphasizing the Midline Angle in degrees (MLA), for a more precise and objective assessment of fetal head position [25]. Despite the advantages of IU, relying on a single parameter or fetal position alone may be insufficient for a full assessment of labor progress [24,28,35]. The recently published Artificial Intelligence Dystocia Algorithm (AIDA) presents a systematic approach to categorizing dystocic labor by integrating four key geometric parameters obtained from IU: the Angle of Progression (AoP), the Head–Symphysis Distance (HSD), the Midline Angle (MLA), and the Asynclitism Degree (AD) [6,7,8] (Figure 1). This multiparameter model, powered by machine learning, has shown high predictive accuracy for delivery outcomes in cases of dystocia [36].

**Figure 2:**
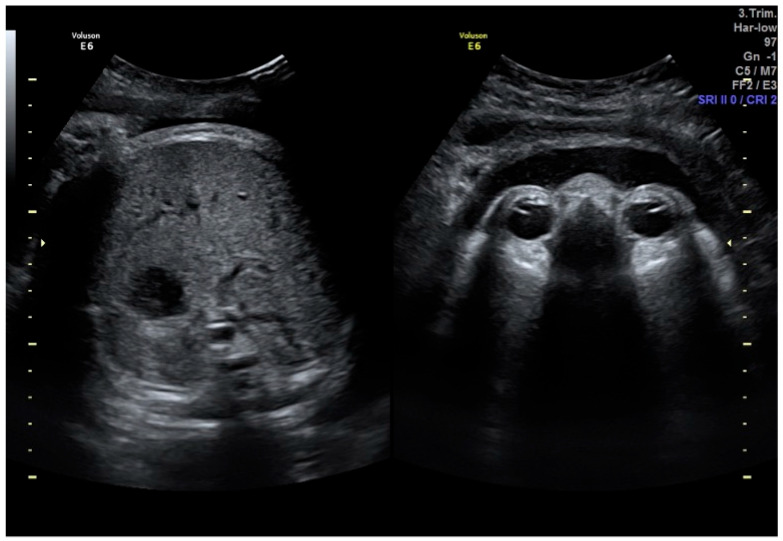
A transabdominal assessment of fetal presentation. The eyes-to-pubis sign (fetal orbits oriented toward the pubis) and a completely posterior fetal spine confirm posterior orientation prior to intervention. These TA adjuncts complement TP metrics (AoP, HSD, MLA, AD) to reduce misclassification of position and station.

Beyond the AoP, HSD, MLA, and AD, several intrapartum ultrasound (IU) metrics have been explored as predictors of progress and instrumental success. Suprapubic Descent Angle (SDA) is a suprapubic transabdominal surrogate of descent that correlates closely (inversely) with the transperineal AoP and shows excellent inter-observer agreement; it can be useful when perineal windows are suboptimal [37]. Head–Perineum Distance (HPD) captures the cranio-perineal gap. Multicenter prospective work suggests that larger HPD values are associated with failed vacuum attempts, while smaller distances support feasibility; in OP-specific cohorts, the HPD has sometimes shown higher predictive performance than the AoP for vacuum success [38,39]. The “head direction” (upward/anterior vs. downward/posterior on TP views) and related vector measures provide a quick visual proxy of engagement and flexion and have been linked to vacuum performance in observational series [24,39]. The Occiput–Spine Angle (OSA) (non-OP) and the Chin-to-Chest Angle (CCA) (OP) quantify head flexion; a smaller CCA (more flexion) and larger OSA have been associated with increased likelihood of vaginal delivery, supporting the clinical importance of attitude/deflexion [26]. Serial assessment during pushing (e.g., ΔAoP) improves prognostic value over static readings and can identify “responders” vs. “non-responders” among apparently similar OP cases [24]. The eyes-to-pubis sign (fetal orbits toward the pubis) and a completely posterior fetal spine on TA views identify true posterior orientation and help discriminate transient posterior positions from truly persistent OP before intervention (Figure 2) [31,32,37].

A narrow subpubic arch angle (SPA) has been associated with a higher risk of OP at birth and operative delivery, although measurement standardization and reproducibility remain issues [40,41]. Deep learning models have achieved automated classification of OA/OT/OP from TP images, suggesting a path to scalable, operator-independent estimation of position and possibly rotation angles [34].

This study applies the AIDA classification to the OP subset of the original AIDA cohort. Only the deterministic classification branch (Branch 1) was used. By contextualizing fetal position with the other three AIDA parameters, we examined whether the multiparametric classification preserves a coherent ordinal risk gradient when restricted to OP positions. The analysis is hypothesis generating and exploratory. It evaluates the descriptive behavior of a pre-specified classification rule within a subgroup of the source cohort. It does not train, test, optimize or validate any predictive machine-learning model. Whether the geometric configuration of the fetal head adds prognostic information beyond rotational status alone in OP labor remains an open question that prospective external validation will need to address.

## 2. Materials and Methods

This investigation was conducted as a focused secondary analysis of data originally collected for the development and validation of the Artificial Intelligence Dystocia Algorithm (AIDA) [6,7,8]. The original retrospective study compiled data from three Italian hospitals spanning from January 2014 to December 2020. The study was conducted in accordance with the Declaration of Helsinki and received approval from the Institutional Review Board (CER 0320). All patient data were anonymized prior to analysis.

The AIDA classification thresholds applied in the present analysis were derived from the original AIDA cohort; the 79 OP cases analyzed here are a subset of that cohort. The present work therefore does not constitute an independent validation of the AIDA system, but a within-cohort subgroup analysis aimed at exploring whether the existing classification preserves a coherent ordinal risk gradient when restricted to OP positions.

The inclusion criteria were nulliparous pregnant women with singleton fetuses in cephalic presentation under neuraxial analgesia who experienced a prolonged second stage of labor, defined as exceeding three hours according to ACOG guidelines [9,10,11]. For the present study, a subset of 79 women with a sonographically confirmed occiput posterior (OP) fetal head position was selected from the original cohort of 135 patients. The exclusion criteria were non-cephalic presentations, multiple pregnancies, abnormal placental implantation, HELLP syndrome, coagulation disorders, uterine hyperstimulation, non-reassuring fetal heart rate, thick meconium, and cephalopelvic disproportion [6,7,8].

Using standard 3.5 MHz transabdominal ultrasound probes, four geometric parameters were measured at the three-hour mark of the second stage. The Angle of Progression (AoP) was defined as the angle between the longitudinal axis of the pubic symphysis and a line tangential to the fetal skull [7,8]. The Head–Symphysis Distance (HSD) was measured as the shortest distance from the inferior margin of the symphysis pubis to the fetal skull [7,8]. The Midline Angle (MLA) was assessed as the angle between the fetal cranial midline and the maternal pelvic anteroposterior diameter (infrapubic line) [7,8]. Finally, the Asynclitism Degree (AD) was measured as the perpendicular distance from the midline to the parietal bone [7].

Each parameter was carefully assessed to capture an accurate representation of the fetal head’s position and progression.

All intrapartum ultrasound examinations were performed by a single experienced obstetrician at each participating center, each with more than ten years of experience in intrapartum ultrasound and all having completed the same standardized AIDA acquisition training protocol. The operator who performed the scan was also the clinician managing the labor, which precluded blinding to clinical context; however, the mode of delivery had not yet been decided at the time of image acquisition and parameter measurement. The four AIDA parameters were measured at a single, pre-specified time point, the three-hour mark of the second stage of labor; serial intra-pushing measurements (e.g., ΔAoP) were not acquired, so the analysis evaluates a static geometric profile. Reproducibility of the four parameters has been documented in independent series. AoP and HSD measured on transperineal ultrasound have shown intraclass correlation coefficients consistently above 0.90 [24,25]. MLA on transabdominal ultrasound is reproducible when the cranial midline is clearly identifiable [24]. AD showed good agreement in our previous AIDA-asynclitism analysis [7]. We did not perform a formal re-assessment of inter- and intra-observer reproducibility within this OP-only subgroup, which is acknowledged among the study limitations.

AIDA comprises two sequential branches: Branch 1 (Classification) uses Decision Tree (DT) algorithms to identify cut-off values for the four parameters and assign cases to five risk classes (0–4) based on color-coded thresholds; Branch 2 (Prediction) applies machine-learning algorithms (SVM, Random Forest, MLP) for probabilistic outcome prediction [6].

These classifications are then aggregated to assign each case to one of five AIDA classes (0–4), where Class 0 represents all parameters being green and Class 4 represents all parameters being red or yellow [6].

The DT algorithm, applied to paired parameter combinations in the original cohort (N = 135), identified cut-off values discriminating between intrapartum cesarean delivery (ICD) and non-ICD outcomes. Each parameter received color-coded risk classification:

Parameter thresholds were as follows:

- AoP: GREEN (non-ICD) 101.5–144.5°; RED (ICD risk) < 101.5° or ≥144.5°;
- HSD: GREEN < 19.5 mm; RED ≥ 19.5 mm;
- MLA: GREEN < 60.5°; YELLOW 60.5–62.0°; RED ≥ 62.0°;
- AD: GREEN < 65.5 mm; YELLOW 65.5–70.5 mm; RED ≥ 70.5 mm.

AIDA class assignment based on unfavorable parameters (RED/YELLOW) was as follows: Class 0 (all GREEN), Class 1 (one unfavorable), Class 2 (two unfavorable), Class 3 (three unfavorable), and Class 4 (all unfavorable).

Outcome Binarization was as follows: four delivery outcomes (spontaneous vaginal, operative vaginal, ICD, ICD after failed OVD) were binarized into NON-ICD (spontaneous + operative vaginal) versus ICD (cesarean ± failed OVD), reflecting the fundamental clinical decision point.

The Decision Tree cut-offs applied were derived from the original AIDA cohort (N = 135) of which the present 79 OP cases form a subset. The four parameters describe position-independent biomechanical relationships (descent, engagement, rotation, lateral tilt), and the cut-offs were not re-fitted on the OP subset; nevertheless, this study evaluates pre-specified thresholds in an OP-enriched portion of the same dataset. The analysis is therefore best interpreted as an internal subgroup evaluation. It does not constitute an independent external validation. A clear risk of optimism and partial circularity exists, which is acknowledged among the study limitations.

The present analysis examined whether the AIDA class assignment alone is associated with the delivery outcome within the OP subgroup, without applying any case-level machine-learning prediction. Previous AIDA reports characterized the predictive performance of Branch 2 in the parent cohort [6,7,8]. Whether the discriminative information already resides at the classification step (Branch 1) and whether this property holds beyond the original cohort remain open questions. Prospective external validation is required to address them.

Statistical analysis used Python 3.12 (pandas, numpy, scipy.stats). Continuous variables were reported as mean ± SD and median (IQR) and categorical variables as frequencies and percentages. Primary hypothesis testing assessed the association between AIDA class (categorical: 0–4) and delivery outcome (binary: vaginal/cesarean) using the chi-square test (null hypothesis: independence; significance *p* < 0.05). Effect size was estimated via Cramer’s V = √(χ^2^/(n × min(r − 1,c − 1))), interpreting V > 0.70 as very strong association, and trend analysis via Spearman’s ρ to evaluate the monotonic relationship between ordinal AIDA class and cesarean outcome, with |ρ| > 0.70 indicating strong monotonic relationship.

Secondary analyses calculated class-specific cesarean rates, PPV for Class 4, and NPV for Class 0. Stratified analyses examined AIDA class distribution across MLA ranges (<60°, 60–74°, ≥75°) to compare multiparametric versus single-parameter (MLA-only) assessment. Parameter distribution analysis identified which geometric parameters were most commonly unfavorable in OP positions.

This approach prioritizes association testing and effect size estimation. These methods are appropriate for evaluating a pre-defined classification framework. The development of new predictive models was outside the scope of this analysis.

## 3. Results

### 3.1. Study Population and Overall Outcomes

Among 135 nulliparous patients, 79 (58.5%) had OP positions. The maternal age was 30.2 ± 4.7 years and gestational age 39.6 ± 1.1 weeks, all with prolonged second stage (≥3 h) under neuraxial analgesia; the MLA ranged from 39 to 90° (mean 63.4°, median 66.0°). There were 23 vaginal deliveries (29.1%) and 56 cesarean deliveries (70.9%). The 70.9% cesarean rate exceeded the institutional baseline for nulliparous OA positions (15 to 20%). This pattern is consistent with the role of OP as an operative delivery risk factor described in the literature.

### 3.2. AIDA Classification and Delivery Outcomes

The distribution of delivery outcomes across AIDA classifications is presented in Table 1. A highly significant association was observed between AIDA class and delivery outcome (χ^2^(4) = 57.49, *p* < 0.001), with a very large effect size (Cramer’s V = 0.853) and a strong monotonic relationship (Spearman’s ρ = 0.738, *p* < 0.001). AIDA class was associated with delivery outcomes in this sample. An increasing cesarean rate was observed across classes.

Statistical significance was as follows: χ^2^ (4) = 57.49, *p* < 0.001; Cramer’s V = 0.853 (very large effect); Spearman’s ρ = 0.738, *p* < 0.001. AIDA class was associated with delivery outcomes in this sample. An increasing cesarean rate was observed across classes.

The extreme AIDA classes showed complete outcome separation in this sample: Class 0 (0/16 cesareans; 95% Wilson CI 0.0–19.4%) and Class 4 (14/14 cesareans; 95% Wilson CI 78.5–100.0%). The wide intervals around these point estimates reflect the small number of cases per class and preclude a claim of perfect discrimination on the basis of these data alone.

Intermediate classes showed a dose-response pattern in this sample (Class 1: 42.9%; Class 2: 88.2%; Class 3: 96.0%).

### 3.3. Parameter Distribution Patterns

MLA distribution in OP cases was as follows: GREEN (<45°) 6 cases (7.6%)—all achieved Class 0 and vaginal delivery; YELLOW (45–59°) 24 cases (30.4%)—distributed across Classes 0–3; RED (≥60°) 49 cases (62.0%)—despite unfavorable rotation, 4 cases (8%) achieved vaginal delivery when compensated by a favorable AoP, HSD, AD.

Cases with RED MLA and GREEN values for the other parameters achieved vaginal delivery in this sample (Classes 0 to 1). Cases with favorable MLA and multiple RED parameters required cesarean (Classes 3 to 4). The within-sample data are consistent with the hypothesis that rotation alone may be insufficient to predict outcomes.

### 3.4. AIDA Classification Across MLA Ranges

To further evaluate the added value of multiparametric assessment over single-parameter rotation evaluation, the distribution of AIDA classes was examined across three MLA ranges, as presented in Table 2.

Within each MLA range, the AIDA classification was associated with heterogeneous within-sample outcomes. For favorable rotation (MLA < 60°), observed cesarean rates ranged from 0 to 100% across classes. For moderate malrotation (60 to 74°), Classes 0 and 1 achieved 43 to 67% vaginal delivery despite concerning rotation. For severe malrotation (≥75°), only Class 1 achieved vaginal delivery. The MLA alone produced a three-level stratification (37%/88%/100%). The AIDA classification produced a finer-grained within-sample risk distribution.

### 3.5. Comparison of Classification Approaches

Table 3 summarizes the performance characteristics of three classification approaches for OP positions: the traditional binary approach, MLA-based spectrum, and the multiparametric AIDA system.

Within this subgroup, the AIDA showed a complete outcome separation in extreme classes of the present sample (Class 0 and Class 4) accompanied by wide Wilson confidence intervals reflecting the small per-class numbers so that no claim of perfect sensitivity or specificity is made, a five-category risk gradient with a strong monotonic trend, and the descriptive identification of favorable OP profiles (Class 0, n = 16) that progressed to vaginal delivery despite posterior orientation. These figures provide a descriptive contrast across classification strategies and do not constitute a formal comparative diagnostic-performance analysis; a head-to-head evaluation of the incremental discriminative value of the multiparametric AIDA classification over the MLA alone (or simpler bivariate combinations) is presented in Section 3.6 as an exploratory comparative analysis, with the caveat that external prospective validation in independent cohorts remains necessary.

### 3.6. Comparative Discrimination of AIDA Versus MLA-Only

To formally evaluate the incremental discrimination provided by the multiparametric AIDA classification over single-parameter MLA assessment, we compared the two approaches using c-statistic, integrated discrimination improvement (IDI), and net reclassification improvement (NRI), with bootstrap-derived 95% confidence intervals (2000 resamples). Predicted cesarean probabilities for the IDI and NRI calculations were obtained from logistic regression models fitted with each predictor (AIDA class as the ordinal score 0–4, MLA as a continuous variable in degrees).

**Figure 3:**
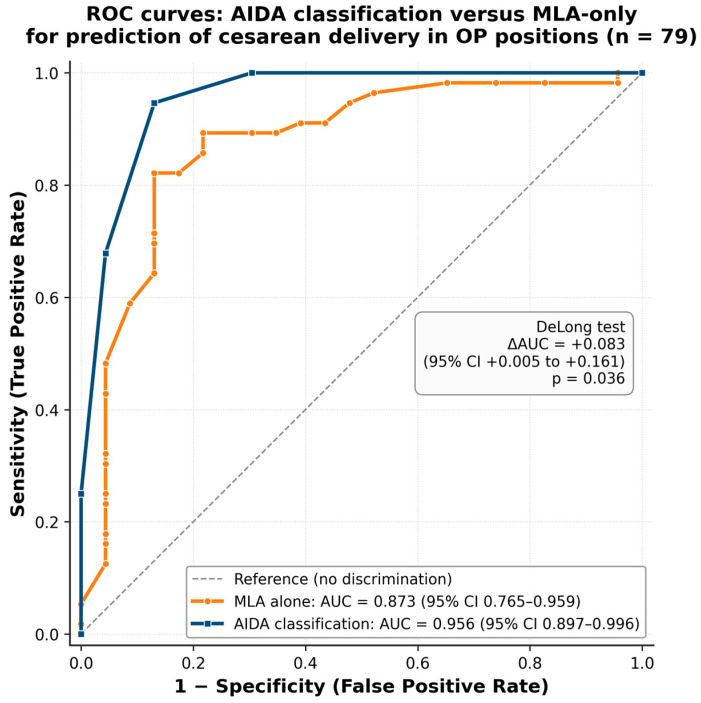
Receiver operating characteristic (ROC) curves comparing AIDA classification (blue) and the MLA alone (orange) as predictors of cesarean delivery in occiput posterior positions during a prolonged second stage of labor (n = 79). AIDA classification yielded an area under the curve (AUC) of 0.956 (95% CI 0.897–0.996), compared with 0.873 (95% CI 0.765–0.959) for the MLA. The difference of +0.083 (95% CI +0.005 to +0.161) was statistically significant by the DeLong test (*p* = 0.036). Bootstrap confidence intervals were derived from 2000 resamples. The dashed diagonal line represents the reference line of no discrimination (AUC = 0.5).

The AIDA classification yielded an area under the ROC curve of 0.956 (95% CI 0.897–0.996), compared with 0.873 (95% CI 0.765–0.959) for the MLA alone. The absolute difference of +0.083 (95% CI +0.005 to +0.161) was statistically significant by DeLong test (z = 2.092, *p* = 0.036), and the AIDA produced a higher AUC than MLA in 99.2% of bootstrap resamples. The integrated discrimination improvement was IDI = +0.266, 95% CI +0.069 to +0.430, *p* < 0.001), with average predicted cesarean probability rising by 7.7 percentage points among cesarean cases and falling by 18.9 percentage points among vaginal cases when the AIDA replaced the MLA as the predictor. The continuous net reclassification improvement was +1.08 (95% CI +0.22 to +1.53). Using clinically meaningful risk thresholds (low < 30%, intermediate 30–70%, high ≥ 70%), the categorical NRI was +0.36, driven by 9 of 56 cesarean cases being correctly upgraded to a higher risk category and 8 of 23 vaginal cases being correctly downgraded. Calibration was acceptable for both models (Hosmer–Lemeshow *p* = 0.39 for both). The full set of comparative discrimination metrics is reported in Table 4, and the corresponding ROC curves for the two predictors are shown in Figure 3.

These analyses indicate that AIDA classification provides a statistically significant gain in discrimination over MLA-only assessment (AUC +0.083, *p* = 0.036). The principal incremental value emerges in reclassification metrics rather than in marginal AUC. The multiparametric framework correctly upgrades cesarean cases and downgrades vaginal cases that the MLA alone would misclassify, supporting the hypothesis that the geometric configuration of the OP fetus (rather than rotation alone) carries within-sample prognostic information.

Two methodological limits bound this interpretation: the comparison is internal (Decision Tree thresholds were derived on the parent cohort that includes the present OP cases, so the AIDA AUC is subject to optimism bias), and the absolute AUC gain is small (+0.08) over an MLA baseline that already discriminates well (AUC 0.87). External validation in independent prospective cohorts is required before any conclusion on clinical utility.

### 3.7. Reconceptualizing the OP Spectrum

Based on our findings, the AIDA describes OP along a geometric risk gradient rather than a purely rotational classification. Three distinct outcome strata emerged from the data and are summarized below as descriptive observations of the present subgroup; their clinical management implications are deferred to Section 4.5 and remain hypothesis generating.

Favorable OP positions (Class 0, n = 16) were characterized by the MLA typically below 50°, AoP above 120°, HSD below 30 mm, and AD below 15 mm. All cases in this group achieved vaginal delivery in this sample. The geometric profile is consistent with partial rotation already achieved or in progress, with the fetal head well positioned for continued descent.

Intermediate-risk OP positions (Classes 1–2, n = 24) showed an MLA between 40° and 75° with a mixed parameter profile and one to two unfavorable parameters. Outcomes varied considerably in this subgroup: Class 1 had a 43% observed cesarean rate, while Class 2 had an 88% observed cesarean rate. The geometric pattern is consistent with partial compensation insufficient to overcome the overall configuration.

High-risk OP positions (Classes 3–4, n = 39) typically showed an MLA at or above 60°, multiple unfavorable parameters, low descent and engagement, marked asynclitism, and no compensatory pattern. Observed cesarean rates were 96–100% in this sample. The geometric profile is consistent with cumulative constraints to spontaneous progression.

### 3.8. Classification Performance: Focus on Risk Stratification

This study evaluated Branch 1 (classification framework based on predefined geometric thresholds) without applying Branch 2 (machine-learning prediction algorithms). Previous AIDA reports across general dystocia and specific malpositions described high prediction performance for extreme classes and >90% overall accuracy in the original cohort. The present analysis addressed whether classification alone, based solely on objective geometric thresholds without ML prediction, retains a coherent risk gradient when applied to OP positions within the same source cohort. Complete outcome separation in the extreme classes (Classes 0 and 4), a very large effect size (V = 0.85), and a clear monotonic gradient (ρ = 0.74) are consistent with the hypothesis that the classification framework alone can support meaningful risk stratification within this subgroup. These observations are descriptive and within-cohort and should be regarded as hypothesis generating regarding the framework’s potential applicability in resource-limited settings where intrapartum ultrasound is available but advanced computing is not. They do not constitute independent validation, which requires prospective external testing.

## 4. Discussion

### 4.1. Principal Findings

This secondary analysis examined 79 occiput posterior cases from a cohort of 135 nulliparous women with prolonged second-stage labor. The findings are consistent with the hypothesis that the AIDA classification may identify a more detailed within-OP risk stratification than fetal position assessment alone. The key observations can be summarized as follows.

The extreme AIDA classes showed complete outcome separation in this OP subgroup (Class 0: 0/16 cesareans, 95% Wilson CI 0.0–19.4%; Class 4: 14/14 cesareans, 95% Wilson CI 78.5–100.0%). Given the small number of cases per extreme class, the confidence intervals are wide, and these findings should be interpreted as a strong descriptive signal rather than as evidence of perfect discrimination. The contrast was obtained applying only Branch 1 of the AIDA (the deterministic, threshold-based classification rule), without invoking the probabilistic machine-learning predictors of Branch 2.

A within-sample risk gradient was observed across the intermediate classes. Cesarean rates increased monotonically from Class 0 (0%) through Classes 1 (43%), 2 (88%), and 3 (96%) to Class 4 (100%). This gradient (Spearman’s ρ = 0.738, *p* < 0.001) is consistent with the hypothesis that the AIDA captures a continuous spectrum of obstetric risk. The pattern does not appear to reflect arbitrary categorical groupings.

In this internal comparison, AIDA classification showed preliminary evidence of incremental discrimination over single-parameter evaluation (AUC 0.96 vs. 0.87 for MLA, *p* = 0.036; see Section 3.6). While the MLA alone showed a relationship with cesarean risk (12.5% for MLA < 50° vs. 100% for MLA ≥ 75°), the multiparametric framework added greater discrimination. Within any given MLA range, the AIDA identified subgroups with vastly different outcomes. For example, among cases with the MLA < 60°, cesarean rates ranged from 0% (AIDA Class 0) to 100% (AIDA Class 4).

The observed within-sample patterns are consistent with quantifiable geometric compensation. The cross-tabulation of AIDA classes across MLA ranges (Table 2) showed that favorable values for the AoP, HSD, and AD may compensate for malrotation in some cases. Four cases with an MLA 60 to 74° achieved vaginal delivery when other parameters were favorable (AIDA Classes 0 and 1). Eleven cases with an MLA <60° required cesarean due to unfavorable descent, engagement, or alignment (AIDA Classes 2 to 4).

These findings are consistent with a multiparameter geometric framing of OP labor, in which rotation is one of four complementary geometric factors. Whether this framing should replace position-based assessment as the primary clinical descriptor remains an open empirical question.

### 4.2. AIDA in Context: Comparison with Alternative Intrapartum Ultrasound Metrics

The pragmatic rationale for limiting the AIDA to the AoP, HSD, MLA, and AD is threefold. First, these four parameters span complementary geometric dimensions (descent, engagement, rotation, and lateral tilt), minimizing redundancy while capturing the main mechanical constraints. Second, they show high feasibility and reproducibility across operators and centers [24,25]. Third, the parameters cover complementary clinical questions. The AoP and HSD have been used in the literature to evaluate the feasibility of a vaginal attempt; the MLA and AD have been used to characterize the rotational and asynclitism profile. By contrast, the SDA is a strong surrogate of the AoP and may be used when TP windows are poor [37]; the HPD adds value primarily for vacuum selection [38,39] and CCA/OSA capture attitude/deflexion, which is informative but not always obtainable [26]. Finally, TA confirmation signs (eyes-to-pubis; posterior spine) are decisional adjuncts that reduce misclassification but are not quantitative inputs to the AIDA [23,31]. The literature on combinations of intrapartum-ultrasound variables suggests that multi-variable approaches may add information over any single metric for predicting delivery mode and failed OVD [42], and management frameworks emphasize ultrasound before intervention [5]. Table 5 provides a comparison of these alternative IU metrics, illustrating their respective planes of measurement, clinical utility, evidence base, and limitations.

While each metric captures specific aspects of fetal head position, the AIDA’s integration of four complementary parameters provides a multidimensional geometric assessment. The present analysis is consistent with the hypothesis that this multiparametric approach is not redundant: each parameter contributes unique information that, when combined, provides more detailed risk stratification compared to any single measurement.

### 4.3. Redefining Persistent Occiput Posterior (pOP) in the AIDA Framework

Persistent OP is traditionally defined as posterior position documented at delivery. This definition ignores how the head evolves across the second stage, and it ignores the surrounding geometry. We propose a different definition: pOP as posterior orientation that persists on serial scans during the second stage, together with unfavorable values for all four AIDA parameters: low AoP (poor descent), high HSD (non-engagement, or “false low station” when caput succedaneum or molding are present), posterior MLA, and marked AD (lateral tilt). Sonographic series of OP labors have consistently linked this combination of persistent posterior orientation and unfavorable head station with protracted second stage, failed operative vaginal delivery, and intrapartum cesarean [17,43,44,45].

The present dataset records each case at a single time point, the three-hour mark of the second stage, and does not include serial intra-pushing measurements. The definition we propose here is therefore a conceptual one, drawn from the geometric profiles at that time point. In this dataset, the high-risk end of the OP spectrum (AIDA Classes 3 and 4) was associated with the highest cesarean rates, while Class 0 cases all reached vaginal delivery. Whether this graded pattern identifies a more uniform high-risk subset than the positional label alone and whether it predicts outcomes that matter clinically are questions for prospective studies with serial AIDA measurements across the second stage.

### 4.4. Integration with Existing Literature

Persistent OP position has been identified as a significant risk factor for operative delivery and cesarean section in several large cohorts [1,2,10]. Most of these studies treat OP as a single entity [12,14,23,31,39,44]. Real-world outcome data, however, are heterogeneous: in one large cohort, 27% of persistent OP deliveries were spontaneous [1], suggesting that compensatory geometric factors may modify the prognosis of any given OP case.

The cross-tabulation analysis in our cohort (Table 2) is consistent with this heterogeneity. Cases with moderate malrotation (MLA 60–74°) achieved vaginal delivery when the AoP and HSD were favorable, while cases with more favorable rotation (MLA < 60°) still progressed to cesarean when other geometric constraints were unfavorable. These observations align with research on fetal head flexion and descent, measured through parameters such as the occiput–spine angle and AoP, in predicting labor outcomes [18,40,46,47]. The well-documented inaccuracy of digital vaginal examination in diagnosing malpositions, especially OP [4,5,22,23,31,33], reinforces the role of intrapartum ultrasound as the primary diagnostic modality. Whether AI-based frameworks could extend sonographic data from diagnosis to prognostic stratification is a question for prospective external evaluation [36].

Whether the multiparametric pattern observed here generalizes to other populations and care environments remains to be established.

### 4.5. Clinical Implications and Management Guidance

The clinical implications discussed in this section are formulated as hypotheses generated by this subgroup analysis, not as recommendations for routine clinical use. The present study did not test an AIDA-guided clinical strategy in an interventional design, and therefore, none of the class-specific management considerations that follow can be considered evidence-based recommendations. Each of these implications must be prospectively evaluated, ideally in a randomized design comparing AIDA-guided management with standard care, before any change in clinical practice can be advocated.

#### 4.5.1. Guiding Operative Interventions

Prior to vacuum in OP positions, intrapartum ultrasound is typically used to confirm true OP position through quantitative MLA assessment and transabdominal adjuncts such as the eyes-to-pubis sign and posterior spine visualization. It is also used to assess feasibility through the AoP and HSD and to characterize asynclitism through AD. The literature reports that favorable AoP and HSD values are associated with vacuum success and that larger AD values are associated with vacuum failure [7,47,48,49,50]. These associations are framed at the level of single-parameter cut-offs in the cited literature and are independent of AIDA classification. Prospective multicenter work in OP indicates that vacuum extraction can be successful when engagement is documented (low HSD, adequate AoP) and asynclitism is limited, whereas larger cranio-perineal distances correlate with failed attempts [38,39]. A systematic review of randomized trials reports that adding intrapartum ultrasound before instrumental delivery improves diagnostic precision but does not, on its own, improve composite outcomes [5,38,48,49]. Whether a structured multiparametric framework such as the AIDA could refine pre-vacuum decision-making beyond these single-parameter associations is a hypothesis to be tested prospectively and is not established by the present retrospective subgroup analysis [24,37,39,47,51].

In the present subgroup, observed vaginal-delivery rates were high in Classes 0–1 and very low in Classes 3–4. Recent prospective multicenter work [38,52] demonstrates that vacuum can succeed in 95% of OP cases when performed with ultrasound guidance and appropriate case selection. Whether AIDA class adds information to this established case-selection process, alongside the parameters and adjuncts already used in clinical decision-making, is a hypothesis for prospective interventional testing. The multicenter POP-OUT trial did not show a significant reduction in operative delivery overall [53,54], whereas other RCTs reported shorter second stages or higher conversion to OA in selected profiles [55,56,57]. A meta-analysis of RCTs [58] concluded little to no overall effect on spontaneous vaginal delivery, with modest time benefits in OP subgroups and probable influence of operator skill and case selection. These results suggest that the benefit of manual rotation, if any, is contingent on case selection rather than on routine application [59]. Table 6 summarizes the available randomized trials and evidence syntheses on this topic.

Whether AIDA-guided selection criteria for manual rotation would alter outcomes is a hypothesis that can only be tested in prospective trials with predefined patient-selection rules and predefined endpoints. The within-sample class composition observed here suggests that profiles with isolated malrotation but otherwise favorable geometry might respond differently to manual rotation than profiles with multiple unfavorable parameters. Within the present cohort, such isolated profiles were rare (only 2 cases of AIDA Class 2 with an MLA at or above 60°). In multi-unfavorable profiles, malrotation is one of several geometric constraints, and the present data cannot establish whether rotation alone would alter the eventual mode of delivery.

#### 4.5.2. Objective Risk Description and Structured Communication

In a clinical area often shaped by subjective qualitative assessment, the AIDA classification could provide quantifiable, reproducible descriptors of fetal head geometry. Within this subgroup, observed delivery outcomes varied across classes: Class 0 had 16/16 vaginal deliveries, Classes 1 and 2 had cesarean rates of 43% and 88%, and Classes 3 and 4 had cesarean rates of 96% and 100%, respectively. If confirmed prospectively, this graded pattern could support more detailed prognostic communication than a categorical OP/non-OP label. Earlier identification of AIDA-RED profiles (Classes 3–4) at the three-hour mark could also be relevant to the well-established complications of prolonged second stage, including chorioamnionitis, postpartum hemorrhage, perineal trauma, neonatal acidemia, and birth-trauma sequelae [1,10,16,60]. The potential benefit would lie in informing the timing of decision-making, not in mandating any specific course of action. Whether AIDA-class-stratified management would yield different maternal or neonatal outcomes than current standard care and whether it would introduce new harms such as over-triage to cesarean is unknown and must be addressed in interventional studies. Whether this structured framing also translates into measurable improvements in intra-team communication, in shared decision-making with the patient, in training programs, or in medico-legal documentation [36] is a further hypothesis that has not been tested in this study. As with the other clinical implications discussed in this section, these are research directions, not clinical recommendations.

### 4.6. Key Messages

Within this subgroup, fetal position alone did not capture the variability in delivery outcomes. Cases with similar MLA values had markedly different outcomes depending on their overall geometric configuration, with cesarean rates ranging from 0% to 100% within any given rotation range. The integration of the MLA (rotation) with three additional geometric parameters, the Angle of Progression (descent), Head–Symphysis Distance (engagement), and Asynclitism Degree (alignment), provided incremental prognostic discrimination. These observations are consistent with the view that labor outcomes in OP depend on geometric cephalopelvic relationship, of which rotation is one component. All points discussed in this section are working hypotheses for prospective testing, not clinical recommendations.

The AIDA framework comprises two branches: Branch 1 (geometric classification into five risk classes derived from Decision Tree thresholds) and Branch 2 (machine-learning outcome prediction). In this OP subgroup, Branch 1 alone produced a coherent ordinal gradient. Complete outcome separation was observed in the extreme classes of this sample (Class 0: 16/16 vaginal; Class 4: 14/14 cesarean), with monotonic progression across intermediate classes. Wilson confidence intervals were wide, reflecting the small per-class numbers. Whether Branch 1 preserves this stratification in independent prospective settings and whether it would support implementation in environments without machine-learning resources require prospective testing.

AIDA classification showed incremental discrimination over single-parameter rotation assessment (Section 3.6). The data are consistent with a graded spectrum within the OP category, from a favorable subgroup (AIDA Class 0: all 16 cases vaginal) to a high-risk subgroup (AIDA Classes 3–4: 96–100% cesarean rates), with intermediate classes occupying intermediate positions. Whether this descriptive gradient can support tailored management in clinical practice cannot be inferred from a retrospective subgroup analysis and remains a hypothesis to be tested prospectively.

The gradient observed here in OP cases is qualitatively similar to that reported by our group in occiput anterior and occiput transverse subgroups from the same parent cohort. This internal consistency is compatible with the biomechanical rationale underlying the AIDA, that descent, engagement, rotation, and alignment jointly determine how the fetal head fits and descends through the maternal pelvis, but it does not constitute independent evidence of generalizability. Whether the pattern reflects a consistent underlying mechanism or a property specific to this cohort can only be established through prospective external validation in independent populations.

As intrapartum ultrasound is more often used in labor management, frameworks that translate sonographic findings into structured descriptors may support clearer communication of risk. The shift from qualitative statements such as “poor progress” or “arrest of descent” to objective geometric descriptors (for example, AIDA Class 3 OP: AoP 95°, HSD 48 mm, MLA 72°, AD 23 mm) is one such direction. The present subgroup analysis is consistent with this rationale but does not establish the AIDA as a validated tool, which remains an open question for prospective research.

### 4.7. Study Limitations

The present analysis carries several limitations that bear directly on how the findings should be read. Data were collected retrospectively from a prospective observational cohort, and management decisions were not protocolized according to AIDA classification, so we cannot assess how AIDA-guided management would alter outcomes relative to standard care. The AIDA Decision Tree thresholds were derived from the original cohort (N = 135) from which the present OP subset is drawn. The four parameters reflect position-independent biomechanical relationships, and the cut-offs were not re-fitted on the OP subgroup, but the present analysis cannot be considered an independent external validation, and a risk of optimism exists. Each participating center had a single experienced operator performing the AIDA measurements, who was also the clinician managing the labor. This likely contributed to measurement consistency but precluded blinding of the parameter measurements from the clinical management process. It also introduces a potential anchoring effect: the same individual who quantified the geometric parameters was subsequently involved in the management decision and may have reinforced the observed association between AIDA classification and delivery outcome. Most cases (85%) originated from a single Italian center. The OP subset is small (n = 79), and the intermediate AIDA Class 1 contains only 7 cases. The observed Class 1 cesarean rate (43%, 3/7) is consistent with intermediate risk, but the confidence intervals are wide and limit precision in any class-by-class statement. The cohort was restricted to nulliparous women with singleton cephalic pregnancies, neuraxial analgesia, and a prolonged second stage of labor, recruited from three Italian centers operating under similar clinical protocols. The findings should not be assumed to apply to multiparous women; to labors without epidural analgesia; to non-cephalic or non-singleton pregnancies; or to obstetric environments with different baseline cesarean rates, training models, or ultrasound infrastructures. We deliberately focused on the deterministic classification step (Branch 1) and did not apply the machine-learning prediction algorithms of Branch 2 (SVM, Random Forest, Multilayer Perceptron) since the aim was to evaluate the descriptive behavior of the classification step alone. Whether Branch 2 prediction adds incremental value in intermediate-risk OP cases (Classes 1 to 3) remains to be tested. The dataset does not include detailed information on specific interventions attempted (manual rotation, vacuum extraction, oxytocin augmentation), so the interaction between management choices and geometric classification cannot be assessed. The primary outcome was binary, vaginal versus cesarean delivery. Maternal morbidity (perineal trauma, postpartum hemorrhage) and neonatal outcomes (Apgar scores, NICU admission) are clinically important and should be addressed in future work. AIDA parameters were measured at the three-hour mark of the second stage. Earlier assessment, at complete dilation or one hour into the second stage, might enable earlier risk identification but requires validation. Ultrasound measurements in the original AIDA studies showed good inter-rater reliability, but real-world implementation depends on operator training and experience, and performance in less experienced hands requires evaluation.

AIDA classification thresholds were originally generated by a supervised machine-learning algorithm (Decision Tree partitioning). In the present analysis, only the deterministic threshold-based Branch 1 was applied, so no predictive AI model was trained, optimized, or validated in this work.

### 4.8. Future Research Directions

Prospective external validation of the AIDA classification is the most pressing priority before any change in clinical practice can be advocated. An adequately powered multicenter study should recruit consecutive OP cases from heterogeneous obstetric settings, including non-Italian and non-academic centers; apply the AIDA classification as a pre-specified frozen rule, without further threshold adjustment; quantify inter- and intra-observer reproducibility of all four parameters prospectively in the OP-specific setting; expand the outcome set beyond binary delivery mode to maternal morbidity, neonatal outcomes, and patient-reported measures; and ideally test an AIDA-guided management strategy against standard care in a randomized design that also examines cost-effectiveness.

Beyond validation, a number of complementary directions are worth pursuing. Earlier measurement, before the three-hour mark of the second stage, could test whether the AIDA supports proactive labor evaluation. Implementation science studies would clarify barriers and facilitators to real-world adoption. The AIDA could be combined with other labor parameters such as uterine contractility and cervical change dynamics [40] and extended to incorporate the trajectory of the four parameters over time rather than a single time point [46]. Finally, OP-specific machine-learning models trained on OP cases alone could be compared with general AIDA algorithms to determine whether tailoring outperforms the general classifier.

To assess the consistency of the classification across settings, a future validation cohort should also include multiparous women; labors without neuraxial analgesia; and obstetric environments with different baseline cesarean rates, racial and ethnic profiles, and ultrasound infrastructures.

## 5. Conclusions

This focused secondary subgroup analysis of 79 occiput posterior cases supports the hypothesis that within the OP spectrum, the multiparametric AIDA classification may stratify the risk of intrapartum cesarean delivery in more detail than fetal position alone. The four geometric parameters (descent, engagement, rotation, and alignment) jointly produced complete outcome separation in the extreme classes of the present sample. In Class 0, 0/16 cases were delivered by cesarean; in Class 4, 14/14 were, with wide 95% Wilson CIs that reflect the small per-class numbers. A monotonic progression was observed across intermediate classes. This pattern was obtained using geometric classification alone, without machine learning. The framework could potentially be evaluated in settings with intrapartum ultrasound capability, conditional on prospective external validation.

Our findings outline a working hypothesis for prospective testing: that the AIDA class assignment (explainable, threshold-based, and computationally lightweight) may itself function as a bedside risk descriptor, with each class carrying an empirically associated distribution of delivery outcomes. The complete separation at the extremes and the gradient observed here are compatible with this hypothesis but do not, on their own, establish it, nor can a within-cohort subgroup analysis substitute for independent prospective evaluation. The gradient was observed in a clinical context that the obstetric literature recognizes as prognostically heterogeneous, where the conventional binary OP/non-OP descriptor and rotational status by the MLA alone are known to stratify poorly. This reinforces the methodological motivation for the prospective external validation now required. Such validation, with predefined endpoints and explicit assessment of clinical impact, is necessary before any recommendation for routine clinical use. The present analysis does not establish the AIDA as a validated decision-support tool, nor does it support class-specific management recommendations. If validation succeeds, the framework’s reliance on threshold-based classification rather than on a fitted predictive model would carry the further advantage of structural portability to settings without computational infrastructure.

## Figures and Tables

**Table 1:** Delivery outcomes by AIDA classification (N = 79).

AIDA Class	Total n (%)	Vaginal n (%)	Cesarean n (%)	Cesarean Rate
Class 0	16 (20.3%)	16 (100%)	0 (0%)	0%
Class 1	7 (8.9%)	4 (57.1%)	3 (42.9%)	42.9%
Class 2	17 (21.5%)	2 (11.8%)	15 (88.2%)	88.2%
Class 3	25 (31.6%)	1 (4.0%)	24 (96.0%)	96.0%
Class 4	14 (17.7%)	0 (0%)	14 (100%)	100%
Total	79 (100%)	23 (29.1%)	56 (70.9%)	70.9%

**Table 2:** AIDA class distribution across MLA ranges.

MLA Range	Class 0	Class 1	Class 2	Class 3	Class 4	Overall CD Rate
<60° (*n* = 30)	13	4	8	4	1	37%
60–74° (*n* = 33)	3	2	7	15	6	88%
≥75° (*n* = 16)	0	1	2	6	7	100%

**Table 3:** Performance of OP classification systems.

Approach	Classification Criteria	Cesarean Rates	Observed Within-Sample Stratification	Practical Considerations
Traditional Binary	OP vs. non-OP (position only)	71% overall	Simple, widely used	No risk stratification within OP
MLA-Based Spectrum	<60°/60–74°/≥75°	37%/88%/100%	Intuitive, rotation-focused	Overlooks descent, engagement, alignment
AIDA Multiparametric	Classes 0–4 (MLA + AoP + HSD + AD)	0%/43%/88%/96%/100%	Five-class within-sample stratification; complete outcome separation observed in Class 0 and Class 4 of this cohort	Requires ultrasound expertise, 4-parameter assessment

**Table 4:** The comparative discrimination of AIDA classification versus the MLA alone for cesarean delivery in OP positions (n = 79). Confidence intervals derived from 2000 bootstrap resamples except where otherwise stated. AUC, area under the receiver operating characteristic curve; IDI, integrated discrimination improvement; NRI, net reclassification improvement.

Metric	AIDA Class (0–4)	MLA (Continuous)	Difference/Statistic	95% CI	*p*-Value
Area under ROC (AUC)	0.956	0.873	+0.083	+0.005 to +0.161	0.036 (DeLong)
Integrated discrimination improvement (IDI)	-	-	+0.266	+0.069 to +0.430	<0.001
Continuous net reclassification (NRI)	-	-	+1.08	+0.22 to +1.53	-
Categorical NRI (cutoffs 30%, 70%)	-	-	+0.36	-	-
Calibration (Hosmer-Lemeshow)	χ^2^ = 1.90, *p* = 0.39	χ^2^ = 3.04, *p* = 0.39	-	-	-

**Table 5:** Alternative intrapartum ultrasound metrics.

Metric	Plane & How to Measure	What It Captures	Typical Use Case	Evidence Notes	Pitfalls
SDA	TA; angle between pubic axis and head vector	Descent (surrogate of AoP)	When TP AoP not feasible	Strong inverse correlation with AoP; excellent ICC (Kamel 2022, AJOG) [37]	No established threshold for vacuum case-selection decisions
HPD	TP; cranio-perineal distance (mm)	Perineal clearance/feasibility	Pre-vacuum decision	Predicts vacuum failure/success; in OP may show higher predictive performance than AoP (Falcone 2025, AJOG [38]; Bellussi 2019, AJOG MFM [39])	Angle-independent; can be altered by perineal compression/edema
Head direction	TP; upward vs. downward orientation	Proxy of engagement/flexion	With AoP/HSD for OVD choice	Associated with vacuum performance (Bellussi 2019, AJOG MFM [39])	Qualitative; operator-dependent
CCA/OSA	TA/TP; flexion angles (°)	Attitude/deflexion	Risk stratification for VD vs. CS	CCA (OP) and OSA (non-OP) associate with VD (Dall’Asta 2021, AJOG [26])	Not always measurable in late second stage
TA signs (eyes-to-pubis; posterior spine)	TA; orbits to pubis; spine posterior	Confirms true OP/pOP	Pre-intervention confirmation	Helpful adjuncts to TP metrics (Akmal 2004, UOG [22]; Blasi 2010, UOG [23]; Dall’Asta 2024, UOG [32])	Not quantitative; fetal position may fluctuate
SPA	TA pelvic outlet (maternal)	Pelvic outlet opening	Background risk (OP/operative)	Narrow SPA associated with pOP/operative delivery (Ghi 2015, UOG [41]; Eggebø 2016, UOG [40])	Technique-dependent; inter-observer variability

**Table 6:** Randomized trials and evidence syntheses on prophylactic/manual rotation in OP/OT.

Study	Design (n)	Timing/Population	Position Confirmed by US	Primary Endpoint	Main Findings	Notes
Phipps 2021 (AJOG MFM, POP-OUT) [53,54]	Multicenter RCT (per full text)	Early 2nd stage OP	Yes	Operative delivery	No significant overall reduction; trends favor rotation	Operator/learning-curve effects
Verhaeghe 2021 (RCT) [55]	RCT (per full text)	Full dilatation, OP	Yes	SVD	↑ conversion to OA; SVD ↑ not consistent	Selection likely key
PROPOP 2021 (RCT) [56]	Multicenter RCT (per full text)	OP/OT at start of 2nd stage	Yes	Operative delivery	Reduction in OVD/CS in selected profiles	Benefit depends on selection
Graham 2014 (Pilot RCT) [57]	Double-blind pilot (per full text)	OP in 2nd stage	Likely yes	Operative delivery	High OVD both arms; feasibility	Underpowered
Burd 2022 (AJOG MFM) [58]	Meta-analysis of RCTs	OP/OT after full dilatation	Yes	SVD; stage duration	No overall SVD gain; modest ↓ stage time in OP	Heterogeneity
Marguier-Blanchard 2019 (Systematic review) [59]	12 studies	OP in 2nd stage	Mixed	–	Technical success high; recommend selective use	Non-randomized majority

## Data Availability

The original contributions presented in this study are included in the article. Further inquiries can be directed to the corresponding authors.

## Abbreviations

The following abbreviations are used in this manuscript:

ACOG	American College of Obstetricians and Gynecologists
AD	Asynclitism Degree
AIDA	Artificial Intelligence Dystocia Algorithm
AoP	Angle of Progression
CCA	Chin-to-Chest Angle
CD	Cesarean Delivery
CS	Cesarean Section
DT	Decision Tree
HPD	Head–Perineum Distance
HSD	Head–Symphysis Distance
ICD	Intrapartum Cesarean Delivery
ICC	Intraclass Correlation Coefficient
IRB	Institutional Review Board
ISUOG	International Society of Ultrasound in Obstetrics and Gynecology
IU	Intrapartum Ultrasound
MLA	Midline Angle
ML	Machine Learning
MLP	Multilayer Perceptron
NICU	Neonatal Intensive Care Unit
NPV	Negative Predictive Value
OA	Occiput Anterior
OAP	Occiput Anterior Position
OP	Occiput Posterior
OPP	Occiput Posterior Position
OSA	Occiput–Spine Angle
OT	Occiput Transverse
OVD	Operative Vaginal Delivery
pOP	Persistent Occiput Posterior
PPV	Positive Predictive Value
RF	Random Forest
RCT	Randomized Controlled Trial
SDA	Suprapubic Descent Angle
SPA	Subpubic Arch Angle
SVD	Spontaneous Vaginal Delivery
SVM	Support Vector Machine
TA	Transabdominal
TP	Transperineal
VD	Vaginal Delivery
VE	Vaginal Examination

## References

[1] Foggin H.H., Albert A.Y., Minielly N.C., Lisonkova S., Koenig N.A., Jacobs E.N., Cundiff G.W. (2022). Labor and Delivery Outcomes by Delivery Method in Term Deliveries in Occiput Posterior Position: A Population-Based Retrospective Cohort Study. AJOG Glob. Rep..

[2] Barrowclough J., Kool B., Crowther C. (2022). Fetal Malposition in Labour and Health Outcomes for Women and Their Newborn Infants: A Retrospective Cohort Study. PLoS ONE.

[3] Wiafe Y.A., Whitehead B., Venables H., Dassah E.T. (2019). Comparing Intrapartum Ultrasound and Clinical Examination in the Assessment of Fetal Head Position in African Women. J. Ultrason..

[4] Chou M.R., Kreiser D., Taslimi M.M., Druzin M.L., El-Sayed Y.Y. (2004). Vaginal versus Ultrasound Examination of Fetal Occiput Position during the Second Stage of Labor. Am. J. Obstet. Gynecol..

[5] Mappa I., Tartaglia S., Maqina P., Makatsariya A., Ghi T., Rizzo G., D’Antonio F. (2021). Ultrasound vs Routine Care before Instrumental Vaginal Delivery: A Systematic Review and Meta-analysis. Acta Obstet. Gynecol. Scand..

[6] Malvasi A., Malgieri L.E., Cicinelli E., Vimercati A., D’Amato A., Dellino M., Trojano G., Difonzo T., Beck R., Tinelli A. (2024). Artificial Intelligence, Intrapartum Ultrasound and Dystocic Delivery: AIDA (Artificial Intelligence Dystocia Algorithm), a Promising Helping Decision Support System. J. Imaging.

[7] Malvasi A., Malgieri L.E., Cicinelli E., Vimercati A., Achiron R., Sparić R., D’Amato A., Baldini G.M., Dellino M., Trojano G. (2024). AIDA (Artificial Intelligence Dystocia Algorithm) in Prolonged Dystocic Labor: Focus on Asynclitism Degree. J. Imaging.

[8] Malvasi A., Malgieri L.E., Difonzo T., Achiron R., Tinelli A., Baldini G.M., Vasciaveo L., Beck R., Mappa I., Rizzo G. (2025). Artificial Intelligence Dystocia Algorithm (AIDA) as a Decision Support System in Transverse Fetal Head Position. J. Imaging.

[9] (2003). ACOG Practice Bulletin Number 49, December 2003: Dystocia and Augmentation of Labor. Obstet. Gynecol..

[10] (2024). First and Second Stage Labor Management: ACOG Clinical Practice Guideline No. 8. Obstet. Gynecol..

[11] (2015). ACOG Practice Bulletin No. 154: Operative Vaginal Delivery. Obstet. Gynecol..

[12] Iversen J.K., Kahrs B.H., Eggebø T.M. (2021). There Are 4, Not 7, Cardinal Movements in Labor. Am. J. Obstet. Gynecol. MFM.

[13] Cunningham F.G., Leveno K.J., Dashe J.S., Hoffman B.L., Spong C.Y., Casey B.M. (2022). Williams Obstetrics.

[14] Cheng Y.W., Shaffer B.L., Caughey A.B. (2006). The Association Between Persistent Occiput Posterior Position and Neonatal Outcomes. Obstet. Gynecol..

[15] Simkin P. (2010). The Fetal Occiput Posterior Position: State of the Science and a New Perspective. Birth.

[16] Tempest N., Lane S., Hapangama D., UK Audit Research Trainee Collaborative in Obstetrics, Gynecology (UK-ARCOG) (2020). Babies in Occiput Posterior Position Are Significantly More Likely to Require an Emergency Cesarean Birth Compared with Babies in Occiput Transverse Position in the Second Stage of Labor: A Prospective Observational Study. Acta Obstet. Gynecol. Scand..

[17] Barth W.H. (2015). Persistent Occiput Posterior. Obstet. Gynecol..

[18] Malvasi A., Tinelli A., Barbera A., Eggebø T.M., Mynbaev O.A., Bochicchio M., Pacella E., Di Renzo G.C. (2014). Occiput Posterior Position Diagnosis: Vaginal Examination or Intrapartum Sonography? A Clinical Review. J. Matern. Fetal Neonatal Med..

[19] Sherer D.M., Miodovnik M., Bradley K.S., Langer O. (2002). Intrapartum Fetal Head Position I: Comparison between Transvaginal Digital Examination and Transabdominal Ultrasound Assessment during the Active Stage of Labor. Ultrasound Obstet. Gynecol..

[20] Dupuis O., Ruimark S., Corinne D., Simone T., André D., René-Charles R. (2005). Fetal Head Position during the Second Stage of Labor: Comparison of Digital Vaginal Examination and Transabdominal Ultrasonographic Examination. Eur. J. Obstet. Gynecol. Reprod. Biol..

[21] Ridley R.T. (2007). Diagnosis and Intervention for Occiput Posterior Malposition. J. Obstet. Gynecol. Neonatal Nurs..

[22] Akmal S., Tsoi E., Howard R., Osei E., Nicolaides K.H. (2004). Investigation of Occiput Posterior Delivery by Intrapartum Sonography. Ultrasound Obstet. Gynecol..

[23] Blasi I., D’Amico R., Fenu V., Volpe A., Fuchs I., Henrich W., Mazza V. (2010). Sonographic Assessment of Fetal Spine and Head Position during the First and Second Stages of Labor for the Diagnosis of Persistent Occiput Posterior Position: A Pilot Study. Ultrasound Obstet. Gynecol..

[24] Bellussi F., Ghi T., Youssef A., Salsi G., Giorgetta F., Parma D., Simonazzi G., Pilu G. (2017). The Use of Intrapartum Ultrasound to Diagnose Malpositions and Cephalic Malpresentations. Am. J. Obstet. Gynecol..

[25] Nizard J., Haberman S., Paltieli Y., Gonen R., Ohel G., Le Bourthe Y., Ville Y. (2009). Determination of Fetal Head Station and Position during Labor: A New Technique That Combines Ultrasound and a Position-Tracking System. Am. J. Obstet. Gynecol..

[26] Dall’Asta A., Rizzo G., Masturzo B., Di Pasquo E., Schera G.B.L., Morganelli G., Ramirez Zegarra R., Maqina P., Mappa I., Parpinel G. (2021). Intrapartum Sonographic Assessment of the Fetal Head Flexion in Protracted Active Phase of Labor and Association with Labor Outcome: A Multicenter, Prospective Study. Am. J. Obstet. Gynecol..

[27] Eggebø T.M., Hjartardottir H. (2024). Descent of the Presenting Part Assessed with Ultrasound. Am. J. Obstet. Gynecol..

[28] Ghi T., Eggebø T., Lees C., Kalache K., Rozenberg P., Youssef A., Salomon L.J., Tutschek B. (2018). ISUOG Practice Guidelines: Intrapartum Ultrasound. Ultrasound Obstet. Gynecol..

[29] Verma M., Bachani S. (2019). Correlation of Digital Vaginal Examination with Transabdominal Ultrasound to Assess Fetal Head Position during Active Labor. J. South Asian Fed. Obstet. Gynaecol..

[30] Zahalka N., Sadan O., Malinger G., Liberati M., Boaz M., Glezerman M., Rotmensch S. (2005). Comparison of Transvaginal Sonography with Digital Examination and Transabdominal Sonography for the Determination of Fetal Head Position in the Second Stage of Labor. Am. J. Obstet. Gynecol..

[31] Akmal S., Kametas N., Tsoi E., Howard R., Nicolaides K.H. (2004). Ultrasonographic Occiput Position in Early Labour in the Prediction of Caesarean Section. BJOG.

[32] Dall’Asta A., Fieni S., Ghi T. (2024). Real-time Ultrasound Demonstration of Successful Manual Rotation of Fetal Occiput Posterior Position. Ultrasound Obstet. Gynecol..

[33] Bagandanshwa K., Mchome B., Kibona U., Salum I., Mangi G., Masenga G., Kavishe A., Mushi C., Egenberg S., Eggebø T.M. (2025). Factors Contributing to Clinical Misdiagnosis of Fetal Head Position: An Ultrasound Based Cohort Study from Tanzania. J. Matern. Fetal Neonatal Med..

[34] Ramirez Zegarra R., Dall’Asta A., Di Pasquo E., Morganelli G., Falcone V., Lizarraga Cepeda E., Falvo G., Bontempo P., Kiener A.J.O., Fieni S. (2024). Prediction of Persistent Occiput Posterior Position by Sonographic Assessment of Fetal Head Attitude at Start of Second Stage of Labor: Prospective Study. Ultrasound Obstet. Gynecol..

[35] Akmal S., Kametas N., Tsoi E., Hargreaves C., Nicolaides K.H. (2003). Comparison of Transvaginal Digital Examination with Intrapartum Sonography to Determine Fetal Head Position before Instrumental Delivery. Ultrasound Obstet. Gynecol..

[36] Malvasi A., Malgieri L.E., Stark M., Tinelli A. (2024). Dystocia, Delivery, and Artificial Intelligence in Labor Management: Perspectives and Future Directions. JCM.

[37] Kamel R., Negm S., Badr I., Kahrs B.H., Eggebø T.M., Iversen J.K. (2022). Fetal Head Descent Assessed by Transabdominal Ultrasound: A Prospective Observational Study. Am. J. Obstet. Gynecol..

[38] Falcone V., Dall’Asta A., Romano A., Mappa I., Geron Y., Bontempo P., Salluce M., Di Pasquo E., Morganelli G., Di Serio M. (2025). Vacuum Extraction Is Successful in 95% of Cases with an Occiput Posterior Position: The Results of a Prospective, Multicenter Study. Am. J. Obstet. Gynecol..

[39] Bellussi F., Salsi G., Simonazzi G., Youssef A., Cataneo I., Cariello L., Ghi T., Pilu G. (2019). A Simple Sonographic Finding Is Associated with a Successful Vacuum Application: The Fetal Occiput or Forehead Sign. Am. J. Obstet. Gynecol. MFM.

[40] Eggebø T.M. (2016). Re: Narrow Subpubic Arch Angle Is Associated with Higher Risk of Persistent Occiput Posterior Position at Delivery. T. Ghi, A. Youssef, F. Martelli, F. Bellussi, E. Aiello, G. Pilu, N. Rizzo, T. Frusca, D. Arduini and G. Rizzo. *Ultrasound Obstet. Gynecol.*
**2016**; *48*, 511–515. Ultrasound Obstet. Gynecol..

[41] Ghi T., Youssef A., Martelli F., Bellussi F., Aiello E., Pilu G., Rizzo N., Frusca T., Arduini D., Rizzo G. (2016). Narrow Subpubic Arch Angle Is Associated with Higher Risk of Persistent Occiput Posterior Position at Delivery. Ultrasound Obstet. Gynecol..

[42] Skinner S.M., Giles-Clark H.J., Higgins C., Mol B.W., Rolnik D.L. (2023). Prognostic Accuracy of Ultrasound Measures of Fetal Head Descent to Predict Outcome of Operative Vaginal Birth: A Comparative Systematic Review and Meta-Analysis. Am. J. Obstet. Gynecol..

[43] Gardberg M. (1998). Intrapartum Sonography and Persistent Occiput Posterior Position: A Study of 408 Deliveries. Obstet. Gynecol..

[44] Carseldine W.J., Phipps H., Zawada S.F., Campbell N.T., Ludlow J.P., Krishnan S.Y., De Vries B.S. (2013). Does Occiput Posterior Position in the Second Stage of Labour Increase the Operative Delivery Rate?. Aust. NZ J. Obstet. Gynaecol..

[45] Phipps H., Hyett J.A., Graham K., Carseldine W.J., Tooher J., De Vries B. (2014). Is There an Association between Sonographically Determined Occipito-transverse Position in the Second Stage of Labor and Operative Delivery?. Acta Obstet. Gynecol. Scand..

[46] Yano E., Sayama S., Iriyama T., Ariyoshi Y., Akiba N., Ichinose M., Toshimitsu M., Seyama T., Kumasawa K., Nakayama T. (2024). Prediction of Spontaneous Vaginal Delivery in the Prolonged Second Stage Using the Delta Angle of Progression. Am. J. Obstet. Gynecol. MFM.

[47] Ghi T., Maroni E., Youssef A., Morselli-Labate A.M., Paccapelo A., Montaguti E., Rizzo N., Pilu G. (2014). Sonographic Pattern of Fetal Head Descent: Relationship with Duration of Active Second Stage of Labor and Occiput Position at Delivery. Ultrasound Obstet. Gynecol..

[48] Dall’Asta A., Falcone V., Ghi T. (2025). Vacuum Extraction in Occiput Posterior Position: How Ultrasound Improve the Outcome. Am. J. Obstet. Gynecol..

[49] Rizzo G., Mattioli C., Mappa I., Bitsadze V., Khizroeva J., Makatsariya A., D’Antonio F. (2021). Antepartum Ultrasound Prediction of Failed Vacuum-Assisted Operative Delivery: A Prospective Cohort Study. J. Matern. Fetal Neonatal Med..

[50] Sainz J.A., Borrero C., Aquise A., Serrano R., Gutiérrez L., Fernández-Palacín A. (2016). Utility of Intrapartum Transperineal Ultrasound to Predict Cases of Failure in Vacuum Extraction Attempt and Need of Cesarean Section to Complete Delivery. J. Matern. Fetal Neonatal Med..

[51] Henrich W., Dudenhausen J., Fuchs I., Kämena A., Tutschek B. (2006). Intrapartum Translabial Ultrasound (ITU): Sonographic Landmarks and Correlation with Successful Vacuum Extraction. Ultrasound Obstet. Gynecol..

[52] Ghi T., Dall’Asta A., Masturzo B., Tassis B., Martinelli M., Volpe N., Prefumo F., Rizzo G., Pilu G., Cariello L. (2018). Randomised Italian Sonography for Occiput POSition Trial Ante Vacuum (R.I.S.POS.T.A.). Ultrasound Obstet. Gynecol..

[53] Phipps H., Hyett J.A., Kuah S., Pardey J., Matthews G., Ludlow J., Narayan R., Santiagu S., Earl R., Wilkinson C. (2021). Persistent Occiput Posterior Position Outcomes Following Manual Rotation: A Randomized Controlled Trial. Am. J. Obstet. Gynecol. MFM.

[54] Phipps H., Hyett J.A., Kuah S., Pardey J., Ludlow J., Bisits A., Park F., Kowalski D., De Vries B. (2015). Persistent Occiput Posterior Position—OUTcomes Following Manual Rotation (POP-OUT): Study Protocol for a Randomised Controlled Trial. Trials.

[55] Verhaeghe C., Corroenne R., Spiers A., Descamps P., Gascoin G., Bouet P.-E., Parot-Schinkel E., Legendre G. (2021). Delivery Mode After Manual Rotation of Occiput Posterior Fetal Positions: A Randomized Controlled Trial. Obstet. Gynecol..

[56] Blanc J., Castel P., Mauviel F., Baumstarck K., Bretelle F., D’Ercole C., Haumonte J.-B. (2021). Prophylactic Manual Rotation of Occiput Posterior and Transverse Positions to Decrease Operative Delivery: The PROPOP Randomized Clinical Trial. Am. J. Obstet. Gynecol..

[57] Graham K., Phipps H., Hyett J.A., Ludlow J.P., Mackie A., Marren A., De Vries B. (2014). Persistent Occiput Posterior: OUT Comes Following Digital Rotation: A Pilot Randomised Controlled Trial. Aust. NZ J. Obstet. Gynaecol..

[58] Burd J., Gomez J., Berghella V., Bellussi F., De Vries B., Phipps H., Blanc J., Broberg J., Caughey A.B., Verhaeghe C. (2022). Prophylactic Rotation for Malposition in the Second Stage of Labor: A Systematic Review and Meta-Analysis of Randomized Controlled Trials. Am. J. Obstet. Gynecol. MFM.

[59] Marguier Blanchard I., Metz J.-P., Eckman Lacroix A., Ramanah R., Riethmuller D., Mottet N. (2019). Rotation manuelle des variétés postérieures: état des lieux de la littérature en 2019. Gynécol. Obs. Fertil. Sénol..

[60] Bellussi F., Livi A., Cataneo I., Salsi G., Lenzi J., Pilu G. (2020). Sonographic Diagnosis of Fetal Head Deflexion and the Risk of Cesarean Delivery. Am. J. Obstet. Gynecol. MFM.

